# The utility of behavioral activation therapy in addressing emotional problems of two depressed students with borderline intellectual disability: A case study

**DOI:** 10.1002/brb3.3651

**Published:** 2024-08-27

**Authors:** Majid Mahmoud Alilou, Saba Maleki

**Affiliations:** ^1^ Faculty of Education and Psychology, Department of Clinical Psychology University of Tabriz Tabriz Iran

**Keywords:** behavioral activation therapy, borderline intellectual disability, depressed students, emotional problems

## Abstract

**Purpose:**

Intellectual disability is one of the neurodevelopmental disorders. Studies indicated that depression and anxiety are the most prevalent emotional problems among the people with intellectual disability. The aim of this study was to investigate the utility of behavioral activation therapy in addressing emotional problems of two depressed students with borderline intellectual disability.

**Method:**

This study is a single‐subject design with multiple baseline and one month follow‐up. Two students with borderline intelligence underwent behavioral activation therapy for 12 sessions. Beck Depression Inventory‐Second Edition, Beck Anxiety Inventory, Oxford Happiness Inventory and Rosenberg Self‐Esteem Scale were used. Data were analyzed using visual inspection of graphed data, changes in trends, improvement percentage and effect size.

**Findings:**

The findings of this study support the utility of behavioral activation therapy in addressing the emotional problems of two depressed students with borderline intellectual disability.

**Conclusion:**

Behavioral activation therapy has had favorable outcomes in reducing depression and anxiety in depressed students with borderline intellectual disabilities.

## Graphical abstract

**Introduction**: Depression and anxiety are common emotional problems among individuals with borderline intellectual disability, impacting their emotional well‐being and daily life.

**Methods**: This research employs a single‐subject design, incorporating multiple baseline measurements and a one‐month follow up period. Behavioural activation therapy was administered to two students with borderline intellectual functioning, utilizing the Beck Depression Inventory‐Second Edition, Beck Anxiety Inventory, Oxford Happiness Inventory and Rosenberg Self‐Esteem Scale. Data analysis was conducted through visual inspection of graphed data, examining trend changes, calculating improvement percentages, and determining effect sizes.

**Results**: Participants showed improvement in depression and anxiety after the therapy. Engaging in enjoyable activities, which is a core component of behavioural activation, has demonstrated effectiveness.

**Conclusion**: Behavioural activation therapy has shown positive results in reducing depression and anxiety among students with borderline intellectual disabilities.

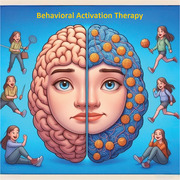

## 1 INTRODUCTION

Intellectual disability or intellectual developmental disorder, which is a neurodevelopmental disorder, includes impairments in intellectual and adaptive functioning, in conceptual, social and practical areas (American Psychiatric Association [APA], 2013). The prevalence of psychiatric disorders in children and adolescents with intellectual disability is significantly higher than children and adolescents without intellectual disability (Buckley et al., 2020; Emerson & Hatton, 2007). Approximately 40% of children with intellectual disability have a diagnosable mental health problem at least twice that of typical children. Emotional problems are two to four times more common in children with intellectual disability than in nondisabled peers (Totsika et al., 2022). Emotional‐behavioral problems are more common in adolescents with borderline intelligence than typical adolescents. These individuals are at risk of poorer mental health (King et al., 2019). Several epidemiological surveys have shown that up to two‐thirds of children and adults with intellectual disability simultaneously develop other mental disorders, several times higher than in people without intellectual disability (Sadock et al., 2015). A study by Einfeld et al. (2011), shows that between 30% to 50% of children and asolescents with intellectual disabilities experience mental disorders. Mood, emotional, depression, and anxiety disorders are the most common psychiatric disorders among individuals with intellectual disability (Emerson & Hatton, 2007; Mazza et al., 2020; Pena‐Salazar, 2020). The results of a meta‐analysis show that the total prevalence of anxiety disorders and depression in children and adolescents who are 6–21 years old with intellectual disability is 7–34% and 3–5% respectively (Buckley et al., 2020).

Research on the treatment of depression in people with intellectual disability has shown the effectiveness of cognitive‐behavioral therapies (Bakken, 2021; Hamers et al., 2018), behavioral therapies that also include behavioral activation (Bakken, 2021; Hamers et al., 2018; Jahoda et al., 2015a, 2015b, 2017; Knight et al., 2019; McCauley et al., 2016; Stuart et al., 2014; Shadan et al., 2021), bright light therapy (Hamers et al., 2018), exercise interventions (John et al., 2020), and the Social Problem Solving Skills Program (Hamers et al., 2018). Other therapies include computer‐aided cognitive‐behavioral therapy, mindfulness‐based cognitive therapy, self‐help intervention, dialectical behavioral therapy, and psychodynamic therapy (Bakken, 2021) and life story intervention (Beernink & Westerhof, 2020). Also, in treatment of anxiety disorders or symptoms in individuals with intellectual disabilities, research has shown the effectiveness of relaxation training programs (Lindsay et al., 1989), exercise interventions (Carraro & Gobbi, 2012; Jacinto et al., 2021), cognitive‐behavioral therapies (Degnan et al., 2018; Fynn et al., 2023; Hronis et al., 2019), behavioral interventions such as graduated exposure and reinforcement (Hagopian & Jennet, 2008), music therapy (De Witte et al., 2020), and finally behavioral activation (McCauley et al., 2016; Stuart et al., 2014) on anxiety symptoms.

Behavioral activation is a short‐term structured therapy for depression. The goal of this treatment is to activate the clients in a way that their rewarding experiences increase in their lives. Although behavioral activation is an independent therapy, it is an important part of cognitive‐behavioral therapy for depression and is similar to other behavioral interventions recently established following the third wave of behavioral and cognitive‐behavioral therapies (Martell et al., 2010).

Since the underlying theory of behavioral activation is associated with that depressive mood can arise from withdrawing from consistent and purposeful activities that have positive consequences, the core of interventions is an attempt to increase the purposeful and personally meaningful activities aimed at overcoming the cycle of avoidance in the patient that causes depression (Knight et al., 2019).

Although behavioral activation guidelines have been used in the treatment of people with clinical depression, the potential of these guidelines in the treatment of anxiety disorders and symptoms is also worth researching (Hopko et al., 2004). The effectiveness of behavioral activation in depression in people without intellectual disability has been very promising. Research shows the effectiveness of behavioral activation in the treatment of depression in typical individuals (Dimidjian et al., 2006; Fernandez‐Rodriguezet et al., 2023; Hopko et al., 2003; Stein et al., 2021) and in the treatment of anxiety in typical individuals (Fernandez‐Rodriguez et al., 2023; Hopko et al., 2003; Stein et al., 2021).

Among psychological therapies, although there are promising advances in cognitive‐behavioral approaches (CBT) for people with intellectual disabilities, behavioral activation may be more accessible to these people than CBT because it is cognitively less challenging (Jahoda et al., 2017) and less than CBT relies on verbal communication and the client's ability to talk about their thoughts and emotions (Jahoda et al., 2015b). Behavioral activation may be particularly appropriate for people with intellectual disabilities who are often socially neglected and have little regular and purposeful activities in their lives (Abbott & McConkey, 2006), and because this approach has fewer communicative demands and focuses on activity, it seems to be an available treatment to these people (Jahoda et al., 2015a).

In relation to the utility of behavioral activation therapy in emotional problems of individuals with intellectual disability, no studies have been done in this regard in Iran and a few studies have been done outside of Iran (Bakken, 2021; Hamers et al., 2018; Jahoda et al., 2015a, 2015b, 2017; Knight et al., 2019; McCauley et al., 2016; Shadan et al., 2021; Stuart et al., 2014).

Therefore, according to the aim of this study, our research hypotheses are that behavioral activation may reduce depression and anxiety and increases happiness and self‐esteem in depressed students with borderline intellectual disability.

## 2 METHOD

### 2.1 Design

This study was an in‐group semi‐experimental (single subject) with multiple baselines. In this study, the baseline staircase design of AB was used which has two stages. Stage A or baseline in which dependent variables are measured, and stage B or intervention in which independent variables are manipulated or entered to determine its impact on dependent variables. Also in stage B, dependent variables were measured. In the present study, depression, anxiety, self‐esteem, and happiness (dependent variables) were measured in baseline (stage A), then independent variable (behavioral activation) was implemented and anxiety, depression, self‐esteem, and happiness of students were measured during and immediately after the intervention (stage B). Also, after one month of follow‐up, dependent variables were measured again.

### 2.2 Subject's history

#### 2.2.1 Subject A

The first subject was a 21‐year‐old girl with borderline intellectual disability who was studying in one of the special schools in the 12th grade in Tehran. She has a married older sister and a younger brother who has dropped out of school (both have normal intelligence), his father died 2 years ago and her mother has substance use disorder. She is from a family whose income is below the federal poverty threshold. The subject is very independent and can handle personal and daily tasks and is also the best student in the school. The subject has a lot of boyfriends and in his own words, she was sexually abused a few years ago. There was a clear instability in her behavior and mood, and many days she complained of feeling sad, unhappy, and tearful. When the concepts and instructions of therapy were explained to her in simple language and with repetition and practice in therapy sessions, she was able to understand them well and also she did the intersession assignments at acceptable level. She had little trouble reading and writing homework during sessions but it only took her a long time to complete the questionnaire. Overall she was very happy with the sessions and loved coming to meetings.

#### 2.2.2 Subject B

The second subject was a 22‐year‐old girl with borderline intellectual disability who was studying in 11th grade in one of the special schools in Tehran. She had been diagnosed with phenylketonuria (PKU) at about age 7. She has a 17‐year‐old younger brother with a normal IQ. Her family self‐reported income is in the lowest income bracket. Her mother was complaining about her lack of movement and overeating and not following her disease‐specific diet. The subject is fully aware of her difference with other people and she is deeply disappointed about that. She is an independent girl who can do her own personal and daily tasks alone and meet her daily needs without the help of her parents, and she also performs very well in school. She is often bored and inactive, she does not do any specific activities during the day, and also she sleeps a lot. She says her mother controls her and compares her to others, causing problems and arguments between them. The subject is irritable and sensitive. She also said that she had experienced love failure. She worked very well during the therapy sessions, understood the simplified concepts explained in the sessions and also was disciplined. She always did the homework and had a good sense of meeting and liked to keep going.

### 2.3 Measures

#### 2.3.1 Beck Depression Inventory‐II

The Beck Depression Inventory‐Second Edition (BDI‐II) was developed in 1996 by Beck, Steer and Brown. This self‐report questionnaire has 21 items, each of which is scored from 0 to 3 and the final score of the subject can be between 0 and 63. Participants were asked to describe their symptoms within the last 2 weeks. Scores between 0 and 13 show minimal depression, 14 and 19 mild depression, 20 and 28 moderate depression, and 29 and 63 severe depression (Beck et al., 1996). Beck et al. (1996) provided evidence that BDI‐II has a suitable validity and reliability for clinical purposes. For example, in a sample of 26 outpatients who had completed this questionnaire before the first and second sessions of cognitive therapy, the 1‐week test–retest reliability was .93 (Beck et al., 1996).

The results of BDI‐II psychometric properties in Iran showed that its items and components are well able to diagnose and evaluate the severity and rate of depression in patients with major depressive disorder (Dobson & Mohammadkhani, 2007). Research also shows that the structure of the Beck Depression Inventory‐II is also appropriate for the individuals with intellectual disability and can be used with confidence in this population (Lindsay & Skene, 2007). The simplified version of this questionnaire was reported to be good and appropriate and alpha coefficient was .90, which indicates the high internal consistency of this questionnaire (Lindsay & Skene, 2007).

#### 2.3.2 Beck Anxiety Inventory

This inventory was developed by Beck, Brown, Steer, and Epstein (1990). Beck Anxiety Inventory (BAI) consists of 21 items of 4 options, each describing common symptoms of anxiety. The respondent is asked to determine how much the symptoms have been bothering him/her during the past week. The choices that the respondent chooses are rated between 0 (at all) and 3 (strongly). Finally, scores are added together to achieve an overall score that can range from 0 to 63 (Beck et al., 1988).

Beck et al. (1988) reported high internal consistency for this scale in a sample of 160 people (Cronbach's alpha = .92). Also, the 1‐week test–retest reliability coefficient was obtained .75. Results of the study by Kaviani and Mousavi (2008) showed that the Persian version of the Beck Anxiety Inventory in the Iranian population had adequate validity (.72), reliability (.83), and internal consistency (alpha = .92). Also, the findings show that BAI possesses adequate reliability in people with intellectual disability (alpha coefficient .91), which indicates high internal consistency of this questionnaire in individuals with intellectual disability (Lindsay & Skene, 2007).

#### 2.3.3 Oxford Happiness Inventory

This inventory was developed in 1989 by Argyle and Lu. It contains 29 items with four options in a format similar to the Beck Depression Inventory. The Cronbach's alpha coefficient was .90. The 1‐week test–retest reliability was .78 and 5‐month test–retest reliability was .67. Also, the external validity of this scale was .43 .The correlation of this questionnaire with Bradburn Positive Affect Scale was .32, with satisfaction (Life Satisfaction Index) was .57, with negative mood and sadness (Beck Depression Inventory) was −.52 (Argyle et al., 1989). In Iran, Oxford Happiness Inventory (OHI) was translated into Persian by Alipour and Noorbala (1999). The internal consistency of the materials of the questionnaire showed that all of its 29 items had a high correlation (from .40 to .73) with the total score. Also, Cronbach's alpha was .93, the split‐half reliability of the test was .92 and test–retest reliability over 3 weeks was .79 (Alipour & Noorbala, 1999). This questionnaire has also been used in individuals with intellectual disability (Khani et al., 2015; Zohoorparvandeh & Jafari, 2017).

#### 2.3.4 Rosenberg Self‐Esteem Scale

Rosenberg Self‐Esteem Scale (RSES) is developed by Morris Rosenberg in 1965, which is made up of 10 items and measures self‐esteem. This scale has a good internal consistency and also test–retest reliability over 2 weeks is reported to be .85 and .88, which indicates the high stability of this scale (Rosenberg, 1979). The Persian version of this scale, unlike the original version, which has 4 options, has two options agree and disagree. In 2005, Mohammadi studied the reliability and validity of RSES in Iran. He analyzed the reliability of this questionnaire through Cronbach's alpha, test–retest and split‐half methods and obtained the reliability coefficients of .69, .78, and .68, respectively. According to the results, the validity and reliability of this scale has been reported to be appropriate in Iran (Mohammadi, 2005). The psychometric properties of this scale have also been investigated in individuals with intellectual disability. The internal consistency of this scale was obtained .64 using Cronbach's alpha coefficient. Also, test–retest reliability over 2 weeks was reported to be .63. In general, the results indicate the moderate validity and reliability of RSES in individuals with intellectual disability (Davis et al., 2009).

### 2.4 Procedure

At first, a meeting session was held for each of the students and initial assessments were conducted at baseline phase. It should be noted that clinical interviews were conducted to diagnose depressive disorders for them before the meeting session. At the meeting session Beck Depression Inventory‐II and Beck Anxiety Inventory were completed by students with borderline intellectual disabilities themselves, so that the questionnaire terms were read and explained to them in a simplified way and they chose an option that was related to their mental condition. Then, Oxford Happiness Inventory and Rosenberg Self‐Esteem Scale were administered on the research sample (as same as two previous questionnaires). In addition, at the meeting session the subjects and their parents were given information about the treatment sessions and goals, and the subjects signed a written consent to announce their readiness to participate in the treatment course. The two subjects were then treated separately for 12 sessions of behavioral activation therapy. The second subject entered the treatment a few days after the first subject. The first subject's treatment sessions were in person and the second subject's treatment sessions were online by using video calls on WhatsApp. Due to adaptation to the needs of students with borderline intellectual disability, the duration of sessions varied from 1 h to 1 h and 30 min. Also, at the beginning of the treatment, sessions were held twice a week, but as the final sessions approached, the sessions were held once a week. The general principles of behavioral activation therapy were based on McCauley et al.’s book in 2016 under the title of “*Behavioural Activation with Adolescents*.” Parts of the books authored by Martell et al. (2010) and Kanter et al. (2009) were also used. However, since the mentioned books are designed for the typical population, in the present study, the concepts and guidelines in these books were simplified for use in individuals with borderline intellectual disability and changes and modifications were made to fit the needs of students with borderline intellectual disability. For example, some of the images presented in McCauley and colleagues’ book were simplified and other images were added to the treatment sessions, to make it easier for students to understand, because the process of learning and treatment is more feasible in individuals with borderline intellectual disability, using visual and objective forms, images, and concepts. Behavioral activation therapy consisted of 12 sessions and the therapist had the option to continue the therapy with more sessions according to the specific needs of these students. Depression, anxiety, happiness, and self‐esteem were measured during the treatment so that subjects were evaluated four times in (B) phase or intervention using the tools used at the baseline phase. The fourth assessment was done as soon as the intervention ended. Also the follow‐up session was held after one month.

### 2.5 Ethical considerations

Regarding the rights of people with borderline intellectual disabilities is one of the most important principles of this research. The Biomedical Ethics Committee of University of Tabriz has approved compliance with ethical considerations in this study. Therefore, ethical principles have been respected for the students and their families. For this purpose, the necessary permits were provided for entering schools and working with students, and the principle of confidentiality of the information was also respected.

After the subjects and caregivers were given general explanations about the treatment process and the goals and decided to participate in the study, they were asked to sign a written consent. It should be noted that the consent text for students was read in simple and understandable language, and they wrote it down and signed it in their own handwriting.

### 2.6 Data analysis

In this study, in order to analyze the data, visual inspection of graphed data, changes in trends, improvement percentage, and effect size were used.

## 3 RESULTS

### 3.1 Scores

In Table 1, scores of Beck Depression Inventory (BDI‐II), Beck Anxiety Inventory (BAI), Oxford Happiness Inventory (OHI), and Rosenberg Self‐Esteem Scale (RSES) in Subjects A and B at baseline, treatment sessions (sess), and follow‐up (f/u) are presented.

**TABLE 1:** Scores of depression, anxiety, happiness, and self‐esteem.

	BDI‐II		BAI		OHI		RSES	
Phases	A	B	A	B	A	B	A	B
Baseline	26	28	31	27	59	54	5	3
Sess 3	13	28	25	22	66	59	7	1
Sess 7	16	28	27	15	60	55	6	1
Sess 10	11	25	30	10	65	58	7	4
Sess 12	14	21	21	16	62	55	7	3
F/U	14	25	27	13	61	56	7	3

BDI‐II = Beck Depression Inventory‐Second Edition. BAI = Beck Anxiety Inventory. OHI = Oxford Happiness Inventory. RSES = Rosenberg Self‐Esteem Scale. Sess = session. F/U = follow‐up.

### 3.2 Changes in trends and slopes

**FIGURE 1:**
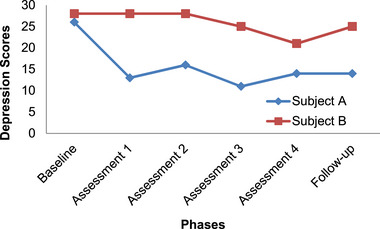
Changes in depression in Subjects A and B.

**FIGURE 2:**
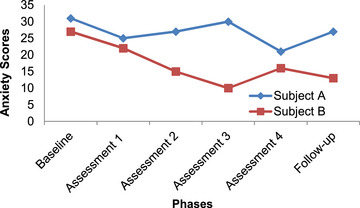
Changes in anxiety in Subjects A and B.

**FIGURE 3:**
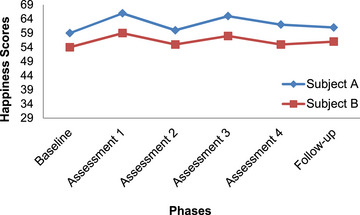
Changes in happiness in Subjects A and B.

**FIGURE 4:**
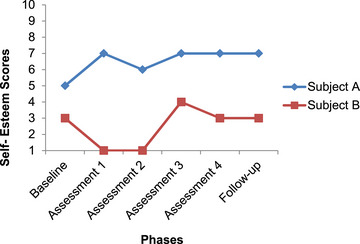
Changes in self‐esteem in Subjects A and B.

In order to analyze the data in this study, the pattern of changes in the scores of subjects is explained using the graphed data. Figures 1, 2, 3, 4 have shown changes in depression, anxiety, happiness, and self‐esteem of Subjects A and B at baseline, treatment, and follow‐up phases.

As shown in Figure 1, changes in depression in Subject A are observed from the beginning of treatment and have a steep slope and then have been almost fixed. Overall, the trend of changes has been decreasing and scores decreased compared to the baseline. Changes in depression in Subject B, which started from the third assessment, were moderate and the slope of changes in this subject was almost minimal. The largest decrease is seen in the final sessions.

In Figure 2, the changes in anxiety level in Subject A are shown, started from the first assessment. The trend of changes is generally low and has a moderate slope; also changes in anxiety score are observed in the follow‐up phase. The changes in Subject B have started since the beginning of treatment and show a steep slope, and the trend of change is declining.

In Figure 3, changes in happiness in Subject A are shown, started from the very first assessment. The slope of change is very variable, and in other words, it has a lot of upheaval. This graph shows an increase, however, insignificant in happiness in Subject A. The changes in trend in Subject B are also very similar to Subject A. In Subject B, changes in happiness level have started since the first assessment, and the slope of these changes is very variable and has many ups and downs. Although happiness has increased slightly in Subjects A and B, but this change is negligible.

Figure 4 shows the pattern of changes in self‐esteem. Changes in the level of this variable in Subject A started from the first assessment. The trend of change is incremental and has a moderate slope. In Subject B, changes in the self‐esteem started from the first assessment, but they showed a decrease in self‐esteem, and only on the third assessment, the self‐esteem score had a sudden increase. The trend of the changes has a steep slope and overall the scores do not show a change in the direction of increase and recovery.

### 3.3 Comparison of the pattern of changes in depression and anxiety in Subjects A and B

Subject A has shown more progress in depression improvement compared to Subject B, but in terms of anxiety, Subject A has experienced less improvement than Subject B. This difference in the pattern of changes can be due to a number of reasons; the first and most important reason may be the unique characteristics of individuals with intellectual disability, which might lead to differences in response to therapy. Another reason could be the virtual nature of the therapy sessions for Subject B that may have affected the transmission of therapeutic concepts. However, more research is needed to gain a deeper understanding of these differences.

### 3.4 Improvement percentage of treatment and follow‐up

In order to investigate the significant changes in the dependent variables, the improvement percentage (IP) is estimated. The scores of subjects in depression (Dep), anxiety (ANX), happiness (HP), and self‐esteem (S/E), and the improvement percentage in treatment (IP in Trt) and improvement percentage in follow‐up (IP in F/U) are presented in Table 2.

**TABLE 2:** Improvement percentage in treatment and follow‐up in Subjects A and B in depression, anxiety, happiness and self‐esteem.

	Subject A				Subject B			
Phases	Dep	Anx	HP	S/E	Dep	Anx	HP	S/E
Baseline	26	31	59	5	28	27	54	3
ASMT 1	13	25	66	7	28	22	59	1
ASMT 2	16	27	60	6	28	15	55	1
ASMT 3	11	30	65	7	25	10	58	4
ASMT 4	14	21	62	7	21	16	55	3
Follow‐up	14	27	61	7	25	13	56	3
IP in Trt	46.15%	32.25%	5.08%	40%	25%	40.74%	1.85%	0%
IP in F/U	46.15%	12.90%	3.38%	40%	10.71%	51.85%	3.70%	0%

Dep = depression. Anx = anxiety. HP = happiness. S/E = self‐esteem. ASMT = assessment. IP in Trt = improvement percentage in treatment. IP in F/U = improvement percentage in follow‐up.

### 3.5 Effect size of treatment and follow‐up

In Table 3, effect size of treatment (ES of Trt) and effect size of follow‐up (ES of F/U) for depression (Dep), anxiety (Anx), happiness (HP), and self‐esteem (S/E) in two subjects are calculated to evaluate the utility of behavioral activation therapy. Also mean of treatment sessions (*M* of Trt sessions), standard deviation of baseline and treatment (*SD* of BL and Trt), mean of treatment and follow‐up (*M* of Trt and F/U), and standard deviation of baseline, treatment and follow‐up (*SD* of BL, Trt and F/U) are presented in Table 3. According to the hypotheses of this study on reducing depression and anxiety and increasing happiness and self‐esteem, the results of Table 3 show that behavioral activation therapy leads to a reduction in depression in Subject A (with treatment effect size of 2.21 and follow‐up effect size of 2.23) and Subject B (with treatment effect size of 0.81 and follow‐up effect of 0.93), a decrease in anxiety in Subject A (with treatment effect size of 1.30 and follow‐up effect size of 1.38) and Subject B (with a treatment effect size of 1.70 and follow‐up effect size of 1.89), an insignificant and negligible increase in happiness in Subject A (treatment effect size of 1.39 and follow‐up effect size of 1.36) and Subject B (treatment effect size of 1.27 and follow‐up effect size of 1.34), increase in self‐esteem in Subject A (treatment effect size of 1.96 and follow‐up effect size of 2.16) and lack of increase in self‐esteem in Subject B (treatment effect size of −0.55 and follow‐up effect size of −0.49). The treatment slightly increased the happiness but this effect was not significant and was considered marginal.

**TABLE 3:** Treatment and follow‐up effect size for depression, anxiety, happiness and self‐esteem in two subjects.

VAR	SUB	BL	*M* of Trt sessions	*SD* of BL and Trt	ES of Trt	F/U	*M* of Trt and F/U	*SD* of BL, Trt & F/U	ES of F/U
Dep	A	26	13.50	5.87	2.21	14	13.60	5.31	2.33
	B	28	25.50	3.08	0.81	25	25.40	2.78	0.93
Anx	A	31	25.75	4.02	1.30	27	26	3.60	1.38
	B	27	15.75	6.59	1.70	13	15.20	6.24	1.89
Hp	A	59	63.25	3.04	1.39	61	62.80	2.78	1.36
	B	54	56.75	2.16	1.27	56	56.60	1.94	1.34
S/E	A	5	6.75	0.89	1.96	7	6.80	0.83	2.16
	B	3	2.25	1.34	–0.55	3	2.40	1.22	–0.49

VAR = variables. SUB = subject. BL = baseline. *M *= mean. Trt = treatment. *SD *= standard deviation. ES = effect size. F/U = follow‐up. *M* of Trt = mean of treatment. *SD* of BL and Trt = standard deviation of baseline and treatment. ES of Trt = effect size of treatment. *M* of Trt and F/U = mean of treatment and follow‐up. *SD* of BL, Trt & F/U = standard deviation of baseline, treatment and follow‐up. ES of F/U = effect size of follow‐up.

In general, hypotheses regarding the utility of behavioral activation therapy in reducing depression, anxiety among depressed students with borderline intellectual disability are supported and the hypotheses about the utility of behavioral activation therapy in increasing happiness and self‐esteem in depressed students with borderline intellectual disability is refuted.

## 4 DISCUSSION

In this study, it can be concluded that, behavioral activation therapy has demonstrated utility in reducing depression and anxiety in two depressed students with borderline intellectual disability. The results of this study in relation to the utility of behavioral activation therapy in reducing depression are consistent with the results of studies by Bakken (2021), Hamers et al. (2018), Jahoda et al. (2015a, 2015b, 2017), Knight et al. (2019), McCauley et al. (2016), Stuart et al. (2014), and Shadan et al. (2021). The mentioned studies are the only few studies that have been performed in people with intellectual disability and their results are completely consistent with the results of the present study. Other studies have also shown the effectiveness of behavioral activation therapy in reducing depression in people without intellectual disability (Chu et al., 2016; Dimidjian et al., 2006; Fernandez Rodriguez et al., 2023; Hopko et al., 2003; Malik et al., 2021; Martin & Oliver, 2019; Ritschel et al., 2016; Stein et al., 2021; Tindall et al., 2017; Valiyan et al., 2017), which results of all these studies are in line with the present study, except that they have been implemented in populations with normal IQ.

The second dependent variable studied in this study is anxiety. As mentioned earlier, the results indicate the utility of behavioral activation therapy in reducing anxiety symptoms of two depressed students with borderline intellectual disability. These findings are consistent with the results of Stuart et al.’s (2014) and McCauley et al.’s (2016) researches. Other than the two studies, no other study has been found to investigate the effect of behavioral activation therapy on anxiety in individuals with intellectual disability, but similar studies have been done on the effectiveness of behavioral activation therapy in reducing anxiety in people without intellectual disability that the results are consistent with the findings of this study (Chu et al., 2016; Fernandez Rodriguez et al., 2023; Hopko et al., 2004, 2006, 2003; Stein et al., 2021; Zemestani et al., 2014).

In this study, happiness was also investigated. As noted in the results, analyses showed that behavioral activation therapy did not lead to a significant increase in happiness in two depressed students with borderline intellectual disability. The results of this study are consistent with the results of the study by Vereenooghe and Westermann (2019). They performed a computerized intervention that included behavioral activation and cognitive reconstruction components in individuals with intellectual disability, but they did not find a statistically significant difference between the two phases of pretest and posttest in subjective well‐being (happiness). On the other hand, some findings of a study by Jahoda et al. (2015a), which implemented behavioral activation therapy on depressed individuals with intellectual disability, are inconsistent with the findings of the present study. In their research, they also showed that the intervention has created positive changes in well‐being (happiness) of intellectually disabled individuals.

No other relevant research was found to measure the effectiveness of behavioral activation therapy on happiness in individuals with intellectual disability. In other studies, the effectiveness of behavioral activation therapy on well‐being and happiness of people without intellectual disability has been investigated, and their results are inconsistent with the findings of this study (Ataie Moghanloo & Ataie Moghanloo, 2015; Ghodrati & Vaziri Nekoo, 2018; Mazzucchelli et al., 2009, 2010).

The last dependent variable studied in this study is self‐esteem. As the data analysis showed behavioral activation therapy did not demonstrate utility in increasing self‐esteem in two depressed students with borderline intellectual disability. The results of Knight et al.’s study in 2019 are inconsistent with the present study. Knight et al. (2019) studied the views and expectations of individuals with intellectual disability who underwent behavioral activation therapy. Study participants stated that intervention had a positive effect on their self‐esteem, self‐confidence, and self‐sufficiency. No other studies were found to directly examine the effect of behavioral activation therapy on self‐esteem in people with intellectual disability.

In people without intellectual disability and with depression, behavioral activation has been shown to be as effective as antidepressants. Its effectiveness among patients with more severe depression is equivalent to or even greater than cognitive‐behavioral therapy, placebos, and other conventional treatments (Ekers et al., 2011; Richards et al., 2016).

Within the realm of psychological treatments, despite the encouraging developments in CBT methods for individuals with intellectual disabilities, behavioral activation might be a more feasible option compared to CBT. This is because it presents fewer cognitive demands (Jahoda et al., 2017) and does not depend as heavily on verbal communication, nor does it require clients to extensively discuss their thoughts and feelings (Jahoda et al., 2015b).

Behavioral activation is a person‐centered behavioral approach that focuses exclusively on “activation” to increase the likelihood of young people encountering naturally rewarding experiences, identifying and reducing barriers to activation, and recognizing avoidance patterns along with expanding alternative coping techniques (McCauley et al., 2016).

Because the underlying theory of behavioral activation is associated with that depressive mood can arise from withdrawing from consistent and purposeful activities that are associated with positive outcomes, the core of interventions is an attempt to increase the targeted and personally meaningful activities with the aim of overcoming the avoidance cycle in the patient that causes depression (Knight et al., 2019). “Activation” is one of the main principles affecting behavioral activation interventions, resulting in greater and more sustained reductions in all avoidance criteria (Fernandez Rodriguez et al., 2023). The results of the studies suggest that behavioral activation may increase neuronal sensitivity to prominent emotional stimuli, such as reward, among anhedonic youth. That is, behavioral activation normalizes the reward neuronal response system in these individuals (Webb et al., 2023).

A study by Hooker et al. (2019) indicates that individuals’ psychological well‐being or happiness is significantly and positively associated with their participation in meaningful and purposeful activities, and since behavioral activation therapy also emphasizes the importance of meaningful and purposeful activities in the person's life, it can be concluded that an important component that leads to the effectiveness of this intervention on psychological well‐being (happiness) is likely to be the activity planning to engage the individual in the targeted and enjoyable activities. While the current study findings indicate that behavioral activation does not lead to an increase in happiness, further research in this area is required.

A research by Dagnan and Sandhu (1999) also indicates that depression significantly and negatively correlates with positive self‐esteem in individuals with intellectual disability.

Although no studies have been found on the effectiveness of behavioral activation therapy on self‐esteem, considering the relationship between self‐esteem and depression, it is valuable to conduct research on the implementation of behavioral activation therapy on self‐esteem.

The present study also has some limitations. The first limitation is the small sample size. Additionally, the research design itself has limitations, which include constraints on statistical inference. Another limitation is that questionnaires which were used in this study were designed and standardized for the typical individuals, due to the lack of access to standardized and appropriate questionnaires suitable for the specific needs of the young people with intellectual disability. Other limitation of this study is relying on self‐report tools. One of the participants' commuting to the therapy sessions was limited, and for this reason, meetings were held online, which is also another limitation of the present study.

## 5 CONCLUSION

The findings of the present study indicate that behavioral activation therapy has demonstrated utility in addressing emotional problems of two depressed students with borderline intellectual disability and in other words, it has had favorable outcomes in reducing depression and anxiety in this population. Therefore, behavioral activation therapy can be used to improve depression and anxiety.

## AUTHOR CONTRIBUTIONS

**Majid Mahmoud Alilou**: Conceptualization; supervision; methodology; formal analysis; project administration; validation. **Saba Maleki**: Investigation; writing—original draft; writing—review and editing; project administration; data curation; visualization; formal analysis.

## CONFLICT OF INTEREST STATEMENT

No conflicts of interest have been declared.

## FUNDING

No external funding was received for the research reported in the paper.

### PEER REVIEW

The peer review history for this article is available at https://publons.com/publon/10.1002/brb3.3651.

## Data Availability

The data that support the findings of this study are openly available in Maleki‐d84c31b2‐5bfa‐49a8‐a82b‐15b5f02674e4 at https://mc.manuscriptcentral.com/brainandbehavior.
